# Correction Notice: Probe-Seq: Method for RNA Sequencing of Specific Cell Types from Animal Tissue

**DOI:** 10.21769/BioProtoc.3961

**Published:** 2021-02-20

**Authors:** Ryoji Amamoto, Mauricio D. Garcia, Jiho Choi, Elizabeth A. Lane, Norbert Perrimon, Constance L. Cepko

**Affiliations:** Departments of Genetics and Ophthalmology, Howard Hughes Medical Institute, Blavatnik Institute, Harvard Medical School, Boston MA 02115, USA; Department of Genetics, Howard Hughes Medical Institute, Blavatnik Institute, Harvard Medical School, Boston MA 02115, USA

After official publication of our protocol in Bio-protocol (https://bio-protocol.org/e3749) we noted an error in the protocol.

In the Materials and Reagents section, the catalog number of Bst DNA Polymerase is "BRP-200", which is completely wrong. Correct catalog number of Bst DNA Polymerase is "BPL-300". Accordingly, we have now corrected as follows:

5. Bst DNA Polymerase (McLab, catalog number: **BPL-300**). Store at -20 °C 

